# A Catalogue of the Medical Plants, Indigenous and Exotic, Growing in the State of New-York, with a Brief Account of Their Composition and Medical Properties

**Published:** 1848-05

**Authors:** 


*A Catalogue of the Medical Plants, Indigenous and Exotic, Growing in the State of New-York, with a brief account of their Composition and Medical Properties.* By Charles A. Lee, M. D. Professor of Materia Medica in Geneva Medical College, and the University of Buffalo, etc. New-York: J. and H. G. Langley. 1848. 8vo. pp. 64.

With characteristic industry, and zeal for the promotion of medical science, Dr. Lee has devoted a considerable amount of labor in the preparation of this catalogue, for which he deserves the thanks of the profession generally, and particularly of the profession of the State of New-York. Dr. L. adopts the natural system of arrangement. which is now fast superseding the complex, but, yet, ingenious classification of Linnæus. We take the following from the preface :—

"The whole number of flowering plants hitherto discovered in this State, according to Prof. Torrey, is about 1450 species. Of these about 1200 are herbaceous, and 150 are ornamental. We have 250 species of woody plants, including eighty that attain the the size of trees. There are also 150 plants that are known to possess medical properties. Of exotics, now naturalized, we have 160 species, many of which have probably been introduced with grain, and other agricultural products from abroad. Of such, are nearly all the weeds that prove so troublesome to the farmer. To the same source, we are also indebted for many of our useful species, as most of our different grasses, which spring up spontaneously on every hand.

Of *Ferns* we have about fifty species belonging to the Flora of the State, some of which are medicinal. The *Male Fern* does not grow within the limits of the State. Our *Mosses*, *Liverworts*, *Lichens and Sea-weeds*, have as yet been but very imperfectly investigated ; though many of them would undoubtedly furnish valuable resources to the medical man. When these have become more fully known, we shall no longer send to Iceland, Ireland, and the East Indies, for mucilaginous mosses, and other remedies of this class. Our *Fungi* are almost innumerable, constituting, probably, at least 3000 species, but few of which have been thoroughly studied. Here is a wide field for such as wish to distinguish themselves by making discoveries in a *terra incognita*. At present our knowledge scarcely suffices to enable us to distinguish such as are poisonous from those which are edible and nutritious. Where is the genius, which is to illustrate this dark region? Where the man, whose name is to be connected with this branch of natural science, in all future time?

It is not to be supposed, that the botany of this state is as yet fully explored. A large majority of our phenogamous plants have undoubtedly been discovered ; but yet we believe that numerous interesting plants of this class remain undetected, besides thousands of the cryptogamic order. The geological features of our State are greatly diversified, and so is its range of temperature ; and the geographical range of plants, being governed by both these circumstances. Already we can number as many species as are found in the whole of New England. But we have mountainous and alpine regions in our State, elevated some 6000 feet above the ocean, which furnish an alpine vegetation, and which remain almost unexplored. We have many plants on our Atlantic borders, as Long Island, which are found no where else in the State ; and the same remark will apply to the Valley of the Hudson, and to our mountainous and western and northern regions. We have many marine plants growing on the borders of our northern lakes, showing that their waters were formerly saline."

